# Hematopoietic Stem Cell Transplantation for DNA Double Strand Breakage Repair Disorders

**DOI:** 10.3389/fped.2019.00557

**Published:** 2020-01-15

**Authors:** Beata Wolska-Kuśnierz, Andrew R. Gennery

**Affiliations:** ^1^Department of Immunology, Children's Memorial Health Institute, Warsaw, Poland; ^2^Translational and Clinical Research Institute, Newcastle University, Newcastle upon Tyne, United Kingdom; ^3^Paediatric Stem Cell Transplant Unit, Great North Children's Hospital, Newcastle upon Tyne, United Kingdom

**Keywords:** artemis deficiency, ataxia-telangiectasia, cernunnos-XLF deficiency, ligase 4 deficiency, Nijmegen breakage syndrome, radiosensitivity

## Abstract

The ubiquitous presence of enzymes required for repair of DNA double strand breaks renders patients with defects in these pathways susceptible to immunodeficiency, an increased risk of infection, autoimmunity, bone marrow failure and malignancies, which are commonly associated with Epstein Barr virus (EBV) infection. Treatment of malignancies is particularly difficult, as the nature of the systemic defect means that patients are sensitive to chemotherapy and radiotherapy. Increasing numbers of patients with Nijmegen Breakage syndrome, Ligase 4 deficiency and Cernunnos-XLF deficiency have been successfully transplanted. Best results are obtained with the use of reduced intensity conditioning. Patients with ataxia-telangiectasia have particularly poor outcomes and the best treatment approach for these patients is still to be determined.

## Introduction

Several DNA repair pathways, which recognize and re-establish non-programmed DNA double-strand breaks (DNA-DSBs) caused by damage from replication errors, ionizing radiation, and/or alkylating agents, have evolved. Ataxia-telangiectasia mutated (ATM) protein and other proteins are activated by DNA-DSBs, leading to phosphorylation of more than 700 proteins which initiate DNA damage signal transduction, cell cycle arrest, and subsequent DNA repair, or activate apoptosis in cells which are catastrophically damaged (1, 2).

As DNA-DSBs directly threaten genomic integrity, the repair processes to correct this damage are indispensable for maintaining genomic structure and reducing the risk of mutagenesis and oncogenesis. Aberrant DNA-DSB repair may lead to localized sequence deviations and genomic information loss. Joining the incorrect pair of DNA ends, which can lead to base deletions, translocations, or inversions, causes more serious damage. Two pathways have evolved to repair DNA-DSBs: homologous recombination (HR) and non-homologous end joining (NHEJ) (3). HR operates predominantly in cells that are dividing and during the S phase and necessitates a homologous template in order to maintain replication accuracy.

NHEJ operates in dividing or non-dividing cells, irrespective of the cell-cycle phase, but is particularly active during stages of the cell cycle when a homologous template is not available. Unlike HR, which accurately repairs DNA-DSB damage, NHEJ is an error-prone process often leading to some loss of DNA information at the site of the DSB.

**Figure 1:**
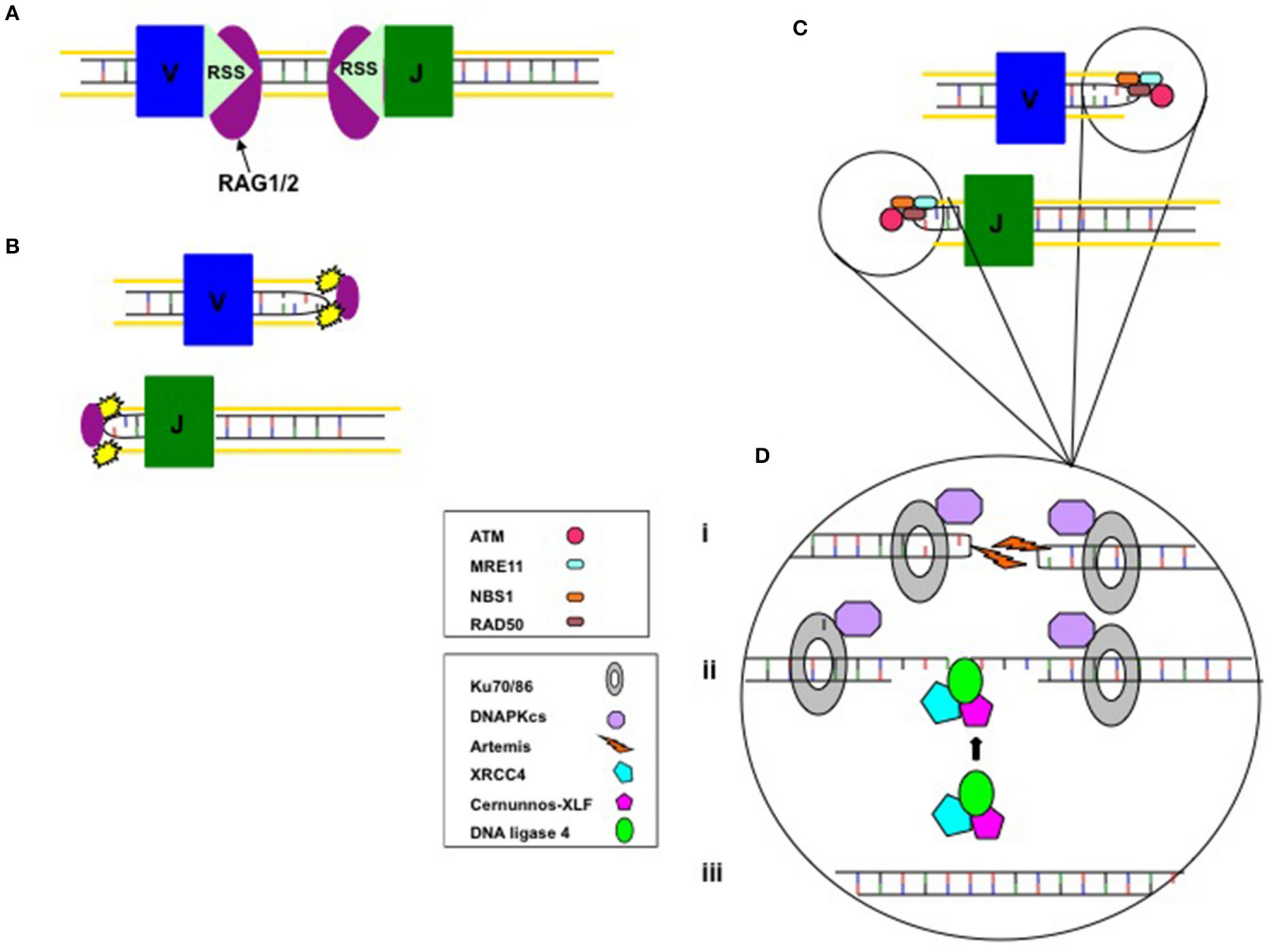
DNA-DSB repair proteins and V(D)J Recombination. **(A)** Lymphoid cell-specific RAG1/2 proteins identify and join to the recombination signal sequences (RSS) that flank V(D)J gene segments. Site-specific DNA-DSB are introduced by the RAG proteins. **(B)** The covalently sealed coding sequence hairpin intermediates are bound together by the RAG complex. **(C)** The MRN complex binds the damaged DNA ends. ATM is activated and commences cell-cycle arrest and recruitment of the repair proteins. **(D)** (i) The Ku70/Ku80 heterodimer binds the coding ends and recruits DNA-PKcs to form the holoenzyme, which recruits Artemis to open the coding end hairpin intermediates by randomly nicking to generate a single-strand break with 3′ or 5′ overhangs. (ii) XRCC4, LIG4, and cernunnos-XLF co-assemble and locate to the DNA ends. The opened coding end hairpin intermediate is modified by nucleotide loss and addition of palindromic and non-templated nucleotides. (iii) Final repair and ligation by the XRCC4/DNA-LIG4/cernunnos-XLF complex. Adapted from Cowan et al. (4).

DNA double-strand break damage initiates sensing of the break, signal transduction and effector function, to commence cell cycle-checkpoint arrest with, or without apoptosis. Repair proteins recruited to damaged DNA sites include those which bind to the DNA break, and occurs in a highly ordered sequence. The MRE11–RAD50–nibrin (MRN) complex initially detects DNA-DSB damage. The broken DNA ends are secured by the MRN complex, which promotes the localized activation of ATM protein. Following ATM activation, several DNA-repair and cell-cycle-checkpoint proteins, including nibrin, are activated, facilitating cell cycle arrest, and DNA repair. More than seven mammalian factors have now been identified as essential NHEJ components. The DNA-binding subunits known as Ku70 and Ku80, with the DNA-dependent protein kinase catalytic subunit (DNA-PKcs or PRKDC), construct the DNA-PK holoenzyme, the role of which is early recognition of DNA-DSBs. The activated DNA-PK holoenzyme recruits other NHEJ proteins including Artemis (DCLRE1C), XRCC4, and DNA ligase 4 (LIG4) to the site of DNA damage (Figure 1). Artemis is phosphorylated by DNA-PKcs, and resolves complex DNA ends. LIG4, XRCC4, and cernunnos-XRCC4-like factor (XLF or NHEJ1) are required for the ligation reaction that rejoins the DNA-DSBs (5).

NHEJ also functions to repair DNA that has been damaged after physiological DNA-DSBs, which are indispensable for development of B- and T-lymphocyte receptor diversity accompanying V(D)J recombination (6). During B- and T-lymphocyte development, DNA-DSBs are introduced during lymphocyte antigen receptor development, immunoglobulin class-switch recombination, and somatic hypermutation. V(D)J recombination is commenced by enzymes coded by recombination-activating gene (RAG)1 and RAG2, defects which cause failure of the V(D)J recombination initiation process, and consequent failure of T- and B-lymphocyte generation leading to T-B- natural killer (NK)+ severe combined immunodeficiency (SCID), or combined immunodeficiency (CID).

There are five other genes in the NHEJ repair pathway that are involved in V(D)J recombination, and in which mutations can cause T-B-NK+ SCID or CID:

- *DCLRE1C*, which codes for an endonuclease (Artemis)
- *LIG4*, which codes for DNA ligase 4
- *PRKDC*, which codes for a phosphokinase [DNA-dependent protein kinase catalytic subunit (DNA-PKcs)]
- *NHEJ1*, which codes for Cernunnos-XLF
- *NBN*, which codes for nibrin, mutated in Nijmegen breakage syndrome, and has a role in the end processing step in NHEJ with Artemis and several other proteins (7–10).

The major distinction between RAG1/2-defective SCID/CID and SCID/CID associated with other deficiencies in the NHEJ pathway is that the NHEJ enzymes are ubiquitously found in all nucleated cells. Therefore, fibroblasts and induced pluripotent stem cells generated from patients harboring these defects display general susceptibility to alkylating agents and ionizing radiation commonly used in conditioning regimens used in allogeneic hematopoietic stem cell transplantation (HSCT). However, defects in RAG1/2 do not display increased susceptibility to these treatments (11–13).

In the non-Artemis defects, development of malignancy is a particularly high risk, with patients predisposed to developing leukemia or lymphoma, often associated with EBV.

## DNA Double Strand Breakage Repair Disorders

### Artemis-Deficiency

Bi-allelic mutations in *DCLRE1C*, located at 10p13 and which encode for Artemis, lead to a wide spectrum of immunodeficiency, accompanied by systemic radiosensitivity, ranging from T-B-NK+ SCID (13) [including Omenn syndrome (14)], through to CID (that may be linked with a predisposition to autoimmunity) (13). Hypomorphic mutations in *DCLRE1C* have been associated with combined immunodeficiency, in which EBV-associated lymphomas have been described (15). Patients with antibody deficiency only are also reported (16). Artemis deficiency is described across the world, but is prevalent in certain populations, particularly Athabascan-speaking native Americans (17), due to a founder mutation. Artemis acts as an endonuclease, activated by the Ku-DNA-PKcs holo-enzyme to process coding sequence hairpin intermediates formed during V(D)J recombination, and is particularly important to resolve complex DNA structures like the heterologous-loop and stem-loop DNA constructions, which contain single-stranded DNA adjacent to double-stranded DNA. Patients with null-mutations present with SCID—the clinical presentation is no different to patients presenting with SCID due to other genetic defects. Unlike other DNA-DSB repair disorders, microcephaly is not a feature. Rare patients are described who have both Artemis deficiency and complete DiGeorge syndrome due to athymic 22q11 deletion (18)—these patients require both hematopoietic stem cell and thymic transplantation. Patients with classic SCID, who may be detected by newborn screening due to absence of TRECs, or presenting with features of combined immunodeficiency should be transplanted.

Historic data have shown that patients with T-B-NK+ SCID have a worse survival outcome than other forms of SCID (19). A landmark three center study examining differences in outcome between Artemis- and RAG-SCID showed no early survival difference between genotypes, but Artemis-deficient patients receiving alkylating agents as part of the preparative cyto-reductive regimen experienced significantly more long-term sequalae including microdontia, growth failure, and autoimmunity (20). However, conditioning without alkylating agents did not permit donor stem cell engraftment, and resulted in poor lymphoid immune reconstitution. More recently, a retrospective study by the Primary Immune Deficiency Treatment Consortium (PIDTC) of North America has reported transplant outcomes in infants with SCID, analyzed by genotype (21). In this analysis, patients with Artemis-deficient T-B-NK+ SCID had a worse survival outcome than those with RAG-deficient T-B-NK+ SCID.

### Nijmegen Breakage Syndrome

Nijmegen breakage syndrome (NBS) is an autosomal-recessive disease caused by mutations of the *NBN* gene on chromosome 8q21. The disease occurs worldwide, but has a high prevalence among Central and Eastern European Slavic populations due to a founder mutation effect. The largest cohort has been diagnosed in Poland (*n* = 118), where all patients carry the same homozygous deletion of five nucleotides (657_661del5) (22).

The Slavic hypomorhic mutation in *NBN* encodes partially functional, truncated p70-nibrin protein, which is a crucial component of the MRN complex involved in DNA double and single-strand breaks repair and in the activation of cell cycle checkpoints.

Almost all patients demonstrate microcephaly at birth, a distinct, dysmorphic facial appearance, becoming more noticeable with age (a prominent midface emphasized by a sloping forehead and receding mandible). Short stature, mild mental retardation, congenital skeletal (clinodactyly, syndactyly), renal, or other abnormalities are also found. In females, premature ovarian insufficiency is observed.

All affected individuals demonstrate a combined immunodeficiency, but of wide severity and clinical manifestation: from “clinically-silent” abnormalities only (disturbed lymphocyte subsets in peripheral blood) to clinically relevant immunodeficiency with hypogammaglobulinaemia. The humoral defect is associated with recurrent, chronic infections, mainly of respiratory tract, leading in some patients to bronchiectasis. About 68 %of patients require immunoglobulin substitution therapy. Surprisingly, there is no correlation between the degree of cellular deficiency (usually decreased number of CD4, CD8 lymphocytes, thymic emigrants, low percentage of naïve cells, increased memory cells, TCRγδ lymphocytes), and severity of infections. Predisposition to opportunistic infections is relatively low and patients do not use routinely antimicrobial prophylaxis. Some patients may be detected on newborn screening for SCID with very low TRECs (23).

The main cause of morbidity and mortality is malignancy, which occurs very early in natural history of disease: most tumors are of hematopoietic origin: lymphoma and leukemia. By age 20 years, over 40% of patients have developed a malignancy, at a median age of 10 years. Patients respond poorly to treatment with a higher incidence of severe or fatal infectious complications during chemotherapy than observed in otherwise healthy children. The high rate of treatment failures and secondary malignancies remain a challenge.

For many years, concerns about the high risk of chemotherapy-induced toxicity and GvHD effectively inhibited attempts to transplant NBS patients. Recently, according to guidelines from the Inborn Errors Working Party of the European Blood and Marrow Transplant society, transplantation is recommended for all NBS patients in first complete remission of lymphoma or leukemia (24). Pre-emptive transplant should be considered before malignancy develops, in patients with clinically relevant immunodeficiency, particularly recurrent or chronic infection despite immunoglobulin therapy or latent viral infections (CMV, EBV). HSCT seems to be the only curative method in patients with immune dysregulation leading to granulomas of the skin and/or other organs (lungs, bones). With increasing numbers of transplanted NBS patients and longer follow up we may be able to answer the most important question: whether the incidence of secondary cancer would be lower than in non-transplanted individuals. There are still not enough data to recommend HSCT in all NBS, even with significant cellular immunodeficiency, but without clinical symptoms of immune deficiency.

### DNA Ligase 4 Deficiency

DNA ligase 4 deficiency, a rare autosomal recessive disorder, is characterized by microcephaly, abnormal dysmorphic (beak-like nose, prominent mid-face, receding forehead, and micrognathia) facial features, combined immunodeficiency, and sensitivity to ionizing radiation. Most patients demonstrate developmental delay. Other described features include bony anomalies and skin conditions. The systemic radiosensitivity confers a susceptibility to malignancy. DNA ligase 4 deficiency is caused by mutations in *LIG4*, which is located on chromosome 13q33–q34. Murine non-sense mutations are embryologic lethal—mutations described in humans are hypomorphic, resulting in significantly impaired, but still functioning NHEJ activity (25).

Little is known of the prevalence of DNA ligase 4 deficiency. Globally <50 cases have been described.

Disrupted V(D)J recombination in DNA ligase 4 deficiency causes severe combined immunodeficiency, Omenn syndrome, and combined immunodeficiency (26–29). Laboratory findings include profound T- and B-lymphocytopenia with varying degrees of hypogammaglobulinaemia, frequently associated with a raised IgM concentration due to defective isotype class switching. Some patients may be detected on newborn screening for SCID with very low TRECs (30). There is increased susceptibility to bacterial, viral, and fungal infection requiring serial hospital admission with an accompanying failure to thrive. Autoimmunity has been described. The risk of development of malignancy is high in patients with DNA Ligase 4 deficiency—to date predominantly tumors of the lympho-reticular system have been described and include T-lymphocyte lymphoblastic leukemia/lymphoma and B-lymphocyte lymphomas (EBV positive or negative)—one case of squamous cell carcinoma has been described (25). The role of HSCT in these patients has yet to be clearly ascertained. For those with low TRECs or features of SCID or CID, HSCT would seem reasonable. Those that develop malignancies are poorly tolerant of chemotherapy and radiotherapy treatment—however the role of pre-emptive HSCT to prevent the development of malignancy has not been established.

### Cernunnos-XLF Deficiency

Autosomal recessive cernunnos-XLF deficiency, due to hypomorphic mutations in *NHEJ1*, located on chromosome 2q35, is extremely rare with <50 reported individuals world-wide. Affected patients share morphological, clinical and immunological features with patients who have DNA Ligase 4 deficiency. Specifically, patients demonstrate microcephaly with distinctive facial dysmorphism (prominent midface emphasized by a sloping forehead and receding mandible) and small stature and developmental delay;—other features described in some patients include boney anomalies (10). Clinical manifestations predominantly include infection due to combined immunodeficiency—autoimmunity and bone marrow failure have also been described. Immunological features that are reported include T- and B-lymphocytopenia and low IgA and IgG in the presence of raised IgM. Recurrent bacterial and opportunistic infections are reported. Lympho-reticular malignancy has rarely been reported in these patients (31, 32). The role of HSCT in these patients has yet to be clearly ascertained.

### Other DNA-DSB Repair Defects

The DNA-binding subunits Ku70 and Ku80 form the DNA-PK holoenzyme, in combination with the DNA-dependent protein kinase catalytic subunit (DNA-PKcs), found on chromosome 8q11.21. The holoenzyme is involved in the early in the recognition of DNA-DSBs, and recruits other NHEJ proteins to the site of damage. To date, no patients have been described with defects in Ku70 or Ku80. Only a handful of patients with DNA-PKcs have been described. Microcephaly has not been described, but too few patients have been recognized to confidently ascertain the classical phenotype, which includes both SCID, CID and autoimmunity (8, 33, 34). Hematopoietic stem cell transplantation has been successfully performed (8, 34)—not enough data are available to recommend any particular approach, although from first principles, patients would be expected to demonstrate a similar response as those with mutations in *DCLRE1C*.

Ataxia telangiectasia-like disorder caused by mutations in *MRE11A*, on chromosome 11q21, is extremely rare, with few patients reported worldwide, predominantly of Arabic background. *MRE11A* encodes for MRE11, part of the MRN complex irresponsible for initial DNA-DSB detection and end-stabilization. Clinical features resemble those of patients with classical ataxia telangiectasia, although progressive cerebellar ataxia occurs later in life with slower progression. Microcephaly has been described in some patients (35). Antibody deficiency is reported, but significant T-lymphocyte immunodeficiency is not described. Lymphoid tumors have not been reported (36, 37). Hematopoietic stem cell transplantation has not been reported for this condition.

Isolated reports of patients with RAD50 deficiency are published (38). RAD50, part of the MRN complex, is encoded by *RAD50*, found on chromosome 5q31.1, and causes a Nijmegen breakage-like syndrome. Patients display radiosensitivity, but immunodeficiency and lymphoid tumors are not reported—hematopoietic stem cell transplantation has not been reported for this condition.

The final stage of DNA-DSB repair requires the ligation complex comprising DNA ligase 4, X-ray repair cross-complementing protein 4 (XRCC4), and XRCC4-like factor (Cernunnos-XLF). XRCC4 protein is encoded by *XRCC4* on chromosome 5q14.2. Several patients have been described with bi-allelic missense mutations in XRCC4 (39–41). Microcephaly, short stature and developmental delay are the predominant features—although there is a marked defect in DNA-DSB repair at a cellular level, V(D)J recombination appears unaffected, implying a separation of function (42). Patients do not present with features of immunodeficiency, and hematopoietic stem cell transplantation has not been reported for this condition.

## Indication for Hematopoietic Stem Cell Transplantation

Each of the genetic defects mentioned above can present with typical features of severe combined immunodeficiency, which is an absolute indication for HSCT. Most, if not all patients with features of significant CID, particularly those of frequent or severe infections despite prophylaxis, significant autoimmunity, lymphoproliferation, or malignancy should be considered for HSCT, preferably before the development of significant end-organ damage or other co-morbidities. Potentially, each of the genetic defects described that can present with immunodeficiency could lead to very low or absent T-lymphocyte receptor excision circles (TRECs) (see chapter on Universal newborn screening for severe combined immunodeficiency), which would be detected by newborn-screening—such patients should be considered for transplantation.

## Approach to Hematopoietic Stem Cell Transplantation for DNA Double Strand Breakage Repair Disorders

Systemic chromosomal fragility and radiosensitivity have important implications for approaching HSCT, particularly with regard to the conditioning agents employed. Although patients with Artemis deficiency generally tolerate fully myeloablative conditioning at the time of transplantation, patients with other DNA-DSB repair defects do not tolerate fully myelo-ablative conditioning regimens. However, a recent report from the PIDTC analyzed patients with Artemis-deficient T-B-NK+ SCID and demonstrated a worse survival outcome than those with RAG-deficient T-B-NK+ SCID (21). The increased mortality appeared not to be related to an excess of infections, suggesting that the intrinsic systemic radiosensitivity may be implicated in the excess mortality. Ideally, for good long term results, these patients need to achieve donor stem cell engraftment, without the use of alkylating agents causing long term sequalae. One possibility in the future may the use of antibody-based conditioning regimens to create space in the marrow niche for donor stem cells, which may lack the long term toxicities (43, 44).

For patients who have other DNA-DSB repair defects described above, myeloablative conditioning is significantly associated with inferior overall survival compared to those who receive a reduced intensity conditioning regimen. A large multi-center series of over 80 patients with LIG4-deficiency, Nijmegen Breakage Syndrome and Cernunnos-XLF deficiency, transplanted for numerous indications including immunodeficiency, severe autoimmunity, bone marrow failure and malignancy, demonstrated that patients receiving a reduced intensity, or Fanconi-based regimen had significantly better survival in the immediate post transplant period than those receiving a fully myelo-ablative conditioning regimen (45). These regimens included standard doses of alemtuzumab or anti-thymocyte globulin serotherapy, but reduced doses of busulphan, cyclophosphamide and melphalan, and reduced to normal doses of fludarabine. The immune related complications resolved with successful HSCT, but other constitutional features such as microcephaly and growth failure were unresolved. Although median follow up was short, no secondary malignancies were described. There was no survival difference relating to the indication for HSCT, or the donor stem cell source. There was a high incidence of acute graft vs. host disease, particularly amongst those receiving myelo-ablative conditioning regimens. These findings have reinforced the recommendation of the Inborn Errors Working Party of the European Blood and Marrow Transplant society to use a reduced intensity conditioning regimen when transplanting these patients (24). There is no place for ionizing radiation in the conditioning regimens for these patients. There are concerns that, as in patients with Fanconi anemia (46), transplantation may predispose to other malignancies in later life—no evidence of this has been documented to date, but long term vigilance will be required for these patients.

An alternative conditioning approach, for some patients at least, may be to use an antibody-based conditioning regimen to achieve myeloid engraftment (43, 44).

### Ataxia Telangiectasia

The ataxia- telangiectasia mutated (ATM) protein is involved in DNA-damage sensing, cell cycle checkpoints and DNA-dsb repair, and is activated upon damage of DNA double strands. Mutations in ATM cause ataxia telangiectasia, a rare autosomal recessive disorder manifest by progressive cerebellar ataxia, oculocutaneous telangiectasia, gonadal sterility, postnatal growth retardation, recurrent sinopulmonary infection which may lead to chronic lung disease, and a high incidence of predominantly lymphoid tumors (47). Few patients have undergone HSCT—the results are generally worse compared to those of patients with other DNA-dsb repair disorders (45). However, most were transplanted for malignancy, which tends to be aggressive and is difficult to treat as patients poorly tolerate the required chemotherapy. Hence it is not clear currently what role HSCT plays in the management of these patients, although reduced intensity conditioning regimens are probably best employed if HSCT is considered. Alternative therapeutic options, which are not yet available clinically, may be the use of antisense oligonucleotides to correct splicing, frameshift and missense mutations and revert absent or unstable protein to partially or fully functional protein (48), or the use of ribosomal read-through agents to overcome premature termination codons, and enable some normal protein expression (49).

## Conclusion

Patients with defects in the DNA-DSB repair pathway experience infections, autoimmunity, bone marrow failure and have an increased risk of lympho-reticular malignancy, particularly associated with EBV infection. The systemic nature of the defect renders them susceptible to chemotherapy-related toxicities, and an increased risk of graft vs. host disease. Best outcomes are obtained with reduced intensity conditioning regimens—antibody-based regimens are likely to reduce toxicity risks further. Long term surveillance is required to ascertain the risk of secondary malignancies. The role of HSCT in patients with ataxia-telangiectasia is to be determined and not currently routinely recommended.

## Author Contributions

BW-K and AG were involved in the inception and design of the review, wrote, and edited the article.

### Conflict of Interest

The authors declare that the research was conducted in the absence of any commercial or financial relationships that could be construed as a potential conflict of interest.
